# Qu-1: a transformation-and regeneration-amenable doubled haploid cell line with a reference genome sequence for genetic and functional studies in *Populus*

**DOI:** 10.48130/forres-0025-0008

**Published:** 2025-04-29

**Authors:** Caixia Liu, Meng Wang, Erqin Fan, Sui Wang, Shuang Li, Pengyue Fu, Yi Liu, Yuhang Liu, Junhui Wang, Heike W. Sederoff, Xiangling You, Chuanping Yang, Ronald R. Sederoff, Su Chen, Guanzheng Qu

**Affiliations:** 1 State Key Laboratory of Tree Genetics and Breeding, Northeast Forestry University, Harbin 150040, China; 2 State Key Laboratory of Utilization of Woody Oil Resource, Central South University of Forestry and Technology, Changsha 410004, China; 3 Key Laboratory of Soybean Biology in Chinese Ministry of Education, Northeast Agricultural University, Harbin 150030, China; 4 State Key Laboratory of Tree Genetics and Breeding, Research Institute of Forestry, Chinese Academy of Forestry, Beijing 100091, China; 5 Department of Plant and Microbial Biology, North Carolina State University, Raleigh, NC 27695, USA; 6 College of Life Science, Northeast Forestry University, Harbin 150040, China; 7 Forest Biotechnology Group, Department of Forestry and Environmental Resources, North Carolina State University, Raleigh, NC 27695, USA

**Keywords:** Qu-1, *Populus*, Doubled haploid, Genome sequencing, Genetic transformation, Ethyl methanesulfonate (EMS)

## Abstract

Plant suspension homogeneous cells are invaluable materials for investigating molecular mechanisms underlying various biological processes. In this study, we established and characterized a doubled haploid cell line from *Populus* *simonii* × *P.* *nigra*, designated as Qu-1. This cell line exhibited high viability and dispersibility under suspension culture conditions and retained the ability to regenerate into whole plants. *K*-mer analysis confirmed the homozygous genome of Qu-1, and a chromosome-level genome assembly was subsequently achieved by using PacBio sequencing. Additionally, we established an efficient transient transformation protocol using PEG-mediated protoplasts and a stable *Agrobacterium*-mediated transformation system for Qu-1. To explore the mutagenesis potential of this cell line, we treated the cell line with ethyl methanesulfonate (EMS) and performed genome resequencing to identify mutation sites. Overall, Qu-1 represents the first poplar cell line with a homozygous genome and is as a powerful tool for molecular biology research in woody plants.

## Introduction

Plant cell suspension cultures provide a valuable source of cell lines that are particularly suitable for genetic and genomic research. Some plant cell lines, such as T87 from *Arabidopsis*^[1]^ and BY-2 from tobacco^[2]^, have been identified and widely used. BY-2 is most widely used due to its rapid proliferation rate coupled with distinctive biological attributes^[3,4]^. This cell line has been used in many studies, such as evaluations of the cell cycle^[5−7]^, cell metabolism^[8,9]^, and abiotic stress responses^[10,11]^. Plant cells are usually recalcitrant to suspension culture, which will typically form obvious callus masses or clusters under suspension culture conditions, despite the efforts from artificial regulation by the addition of phytohormone. Despite extensive efforts, the successful development of suspension cell lines has been achieved in only a small fraction of plant species^[12,13]^. Most of these cell lines however exhibit notable deficiencies, such as loss of cell totipotency, high genome heterozygosity, and the absence of high-quality genome sequences^[14]^. Additionally, although numerous tree species have been studied in molecular research, no well-established suspension cell line derived from trees has been developed^[15−17]^.

High genomic heterozygosity often presents a major challenge for genetic and functional characterization of genes or genome elements in molecular breeding in higher plants, and plant materials with low heterozygosity are highly desirable for researchers. Unlike conventional inbreeding or selfing, haploid induction provides an efficient way to approach homozygosity through chromosome doubling. Haploid and doubled haploid (DH) plants have significantly contributed to numerous studies in plant biology. For example, heterozygosity is a barrier to achieving high-quality genome assembly, whereas haploid or DH plants produced improved genome sequencing, such as that of potato^[18]^, *Eucommia* *ulmoides*^[19]^, and apple^[20]^. *Populus* was the first tree species to be sequenced for its genome^[21]^, and has been used as a model tree species for genetic and molecular research. As a dioecious species, *Populus* undergoes obligate outcrossing, resulting in high levels of heterozygosity. Genome sequencing studies of several poplar species reveal that there are at least more than one million SNPs or small indels within each genome^[21−23]^. Like many other tree species, *Populus* has a prolonged juvenile phase−typically exceeding four years−before reaching reproductive maturity. This extended developmental timeline makes conventional inbreeding approaches to achieve homozygosity highly impractical. As a result, haploid induction followed by chromosome doubling presents a promising alternative. The first report of haploid induction in *Populus* was by Von Kopecky in 1960 using induced parthenogenesis^[24]^, and since then various methods have been used to induce haploid plants of poplar^[25−27]^. However, as noted previously^[28]^, poplar species often exhibit recalcitrance to haploid induction, and to date, high-quality genome sequences and efficient transformation and regeneration systems derived from haploid or doubled haploid *Populus* lines remain limited.

In this study, we report the development of a doubled haploid (DH) cell line from *Populus*, designated Qu-1, which exhibits unique characteristics favorable for suspension culture. We present a high-quality genome assembly of Qu-1 to facilitate genetic and molecular investigations. We anticipate that this resource will serve as a valuable biological system for advancing genetic and molecular research in woody plants.

## Materials and methods

### Plant materials

The Qu-1 callus was cultured on MS medium^[29]^ supplied by 2.0 mg/L 2,4-dichlorophenoxyacetic acid (2,4-D), 1.0 mg/L kinetin (KT), 30 g/L sucrose, and 3 g/L agar. The culture was kept in a dark room at 25 ± 1 °C and subcultured every 15 d.

Qu-1 suspension culture was prepared by taking approximately 1.21 g of callus tissue, dividing it into small aliquots, transferring it into liquid medium (MS + 1.25 mg/L 2,4-D + 0.2 mg/L KT + 50 g/L sucrose), and incubating it at 26 °C with continuous shaking at 110 rpm.

Donor tree (DT, *Populus simonii* × *P. nigra*) and Qu-1 plants were grown in a greenhouse (temperature 25 ± 1 °C, light intensity 1,000−1,500 Lx, 16-h light/8-h dark cycle). The soil is composed of peat moss, vermiculite, and perlite in a ratio of 5:2:1.

Qu-1 plantlets and Ethyl methane sulfonate (EMS) mutant plantlets were cultured in MS medium with the addition of 1.0 mg/L 6-Benzylaminopurine (6-BA) + 0.2 mg/L Indole-3-butyric acid (IBA) + 25 g/L sucrose + 3 g/L gelrite in a tissue culture room (temperature 25 ± 1 °C, light intensity 1,000−1,500 Lx, 16-h light/8-h dark cycle).

### Determination of dispersion and growth rate of Qu-1 suspension cell line

Approximately 1.21 g of Qu-1 callus was transferred into suspension medium (MS + 1.25 mg/L 2,4-D + 0.2 mg/L KT + 50 g/L sucrose), and cultured at 27 °C and 130 rpm in the dark. The suspension cells on the 6^th^ day of culture were taken to a 1.5 mL centrifuge tube, centrifuged at 4,000 rpm to remove the supernatant, and then washed with 0.01 M PBS buffer (8 mM Na_2_HPO_4_ + 137 mM NaCl + 2 mM NaH_2_PO_4_, pH 7.2). The cells were centrifuged at 4,000 rpm to remove PBS. Finally, the cells were resuspended with 1 mL of 0.01 M PBS buffer, and 10 μL of cell suspension was taken to a glass slide, and then the cell morphology and dispersion were observed on an optical microscope. Secondly, the callus of 1.21 g Qu-1 and BY-2 were weighed and transferred to the suspension medium (MS + 2 mg/L 2,4-D + 30 g/L sucrose), cultured at 27 °C, 130 rpm continuous oscillation culture. During the culture process, the concentration of the supernatant of the two cell lines was measured every 4 d. The suspension cells were taken out from the constant temperature shaking incubator, placed on a horizontal operating table, and cultured at room temperature for 20 min. The supernatant (~3 mL) was pipetted into a cuvette with a disposable sterile dropper. The absorbance at OD_600_ was measured using a spectrophotometer, and each measurement was repeated three times. The absorbance was calculated and analyzed with the BY-2 cell line as the control.

### Plant regeneration from Qu-1

Qu-1 callus and regeneration plants were grown in a tissue culture room (temperature 25 ± 1 °C, light intensity 1,000−1,500 Lx, 16-h light/8-h dark cycle). The callus was placed in shoot induction medium (Formulation III: MS + 1 mg/L 6-BA + 0.2 mg/L NAA + 0.05 mg/L TDZ + 25 g/L sucrose + 3 g/L gelrite)^[17]^ and subcultured every 30 d. Vigorously growing shoots 2 cm in length were removed and placed in root induction medium (1/2 MS medium + 0.4 mg/L indole butyric acid (IBA) + 0.02 mg/L naphthalene acetic acid (NAA) + 20 g/L sucrose + 7.47 g/L agar), and whole plants were obtained.

### Qu-1 homozygosity detection

Based on the information of the whole genome resequencing of *P.* *simonii* × *P.* *nigra*, the HaplotypeCaller in GATK was used to detect the variation (SNP/INDEL), and the accurate SNP and INDEL detection were achieved by local recombination of haplotypes. VariantFiltration was used to filter the detection results, FS > 30.0, QD < 2.0, with 35 bases as a window, if there were more than three, the final vcf file was obtained. According to the final vcf file, SNP loci were found and further confirmed by samtools manual review. Two SNP loci were selected in the genome of *P.* *simonii* × *P.* *nigra* and specific primers were designed for the sequence (primers provided in Supplementary Table S1). The length of the product was 250 bp. Subsequently, DT and Qu-1 were used as materials, DNA was extracted by the CTAB^[30]^ method, and PCR was carried out with the above specific primers. The PCR products were sequenced by Sanger sequencing, and the specific sequences were used to compare and analyze whether there were SNP sites (primers are provided in Supplementary Table S1).

### The ploidy of Qu-1 was detected by flow cytometry (FCM)

An appropriate amount of DT and Qu-1 tissue materials were placed in petri dishes, and 1 mL of pre-cooled WPB nucleus extract (0.04 g MgCl_2_·6H_2_O + 0.035 g + EDTANa_2_·2H_2_O + 0.25 g NaCl + 0.095 g Na_2_S_2_O_5_ + 3.15 mL Tirs-HCl (pH7.0) + 500 μL Triton X-100 + 0.5 g PVP-10), and then the materials were quickly chopped with a single-sided blade, and then the mixture suspension was filtered through a 300-mesh filter membrane, collected into a 2 mL centrifuge tube, incubated in a refrigerator at 4 °C for 5 min, followed by centrifugation with 1,000 rpm for 8 min at 4 °C. The supernatant was discarded and the precipitate was added 500 μL PI dye, re-suspended, and placed in a refrigerator at 4 °C for 20 min. The material was loaded into a flow cytometer (Sysmex CyFlow Ploidy Analyser), and 20,000 cells were collected at the same flow rate, voltage, and dye.

### Fluorescence *in situ* hybridization (FISH)

The healthy root tip of a 1 cm long Qu-1 plant was selected as the detection material. The root tip was removed, the surface nutrients and agar were washed with deionized water and then moved into ddH_2_O, incubated at an ice-water mixture (0−4 °C) for 24 h, and then moved into Carnoy's Fluid (3 mL of anhydrous ethanol mixed with 1 mL of acetic acid to obtain 4 mL solution), incubated at 4 °C for 24 h. Then the root tips were washed with 70% ethanol, transferred into ddH_2_O, and incubated at 37 °C for 40 min. Finally, the root tip was moved to the slide, covered with a coverslip, and gently pressed to spread the chromosomes. The coverslip was then carefully removed by freezing the slide in liquid nitrogen for 30 s and gently tapping its edge. Subsequently, the slide was treated with 0.2 M NaOH and 70% ethanol mixed solution at room temperature for 10 min. Subsequently, they were transferred to 70% ethanol (−20 °C) and treated at −20 °C for 1 h, and quickly air-dried. A 10 μL hybridization solution with a probe (TTTAGGG)_20_ was added to each slide and incubated overnight at 37 °C. Finally, each slide was washed three times (5 min each time) in 2 × SSC at 42 °C and stained with 4',6-diamidino-2-phenylindole (DAPI) in VectaShield anti-fading solution. FISH experiments were performed as described^[31,32]^. Microphotographs were taken using a Zeiss LSM700 laser scanning microscope.

### The genome size and heterozygosity of Qu-1 were analyzed by *k*-mer

DNA was extracted from the DT and Qu-1 respectively. After the DNA samples were qualified, the DNA samples were randomly broken into fragments with a length of 300−500 bp by a Covaris ultrasonic crusher. Then the broken fragments were subjected to terminal repair, A tail, sequencing adapter, purification, PCR amplification, and other steps to complete the whole library preparation. Finally, the constructed library was sequenced by MGIseq2000 for PE150 sequencing. The genome feature estimator (GCE, version 1.0.2)^[33]^ was used for *k*-mer analysis of PE library sequencing, and Excel 2019 was used to obtain *k*-mer distribution to evaluate the sample genome size and heterozygosity level.

### Whole genome sequencing and assembly of Qu-1

Firstly, the genomic DNA of Qu-1 was extracted using the CTAB method, and the concentration and quality of DNA were accurately determined by NanoDrop 2000C ultramicro spectrophotometer and Qubit fluorescence meter. Subsequently, the third-generation sequencing library was constructed and high-throughput sequencing was performed using the PacBio Sequel II platform to obtain long-reading data. At the same time, in terms of transcriptome analysis, we used the Biotech General Plant Extraction Kit (BioTeke, RP3301) to obtain the total RNA of Qu-1, and evaluated the quality, concentration, and integrity of RNA by NanoDrop 2000C and Agilent 2100 RNA 6000 Nano. Qualified RNA samples were used to construct full-length transcriptome and common transcriptome libraries. The full-length transcriptome sequencing was performed using the PacBio Sequel II platform, while the second-generation transcriptome sequencing was performed using the MGISEQ-2000 platform. In the assembly process of sequencing data, we used Pb-assembly software^[34]^ to preliminarily assemble PacBio long-read data. To further improve the assembly quality, we used NextPolish^[35]^ and Pilon^[36]^ software, combined with PacBio and NGS^[35−37]^ reads, to calibrate the assembly results. The sequencing work was entrusted to Wuhan Frasergen Information Co., Ltd. (Wuhan, China).

### Hi-C analysis and pseudochromosome construction

The collected fresh callus was treated with 2% formaldehyde and NIB solution under vacuum for 45 min to fix the DNA. The chromatin was cross-linked and digested using the *Mbo* I restriction enzyme. The MGISEQ-2000 platform with a 150 bp PE layout was used to sequence the Hi-C library. The Juicer process was used to analyze the Hi-C data, including indexing using bwa^[37]^ (v0.7.17), finding restriction sites, and then read mapping. 3D-DNA^[38]^ (v180922) was then used for scaffolding, and Juicebox (v1.11.08) was used for manual adjustment to obtain chromosome-level genome sequences^[38,39]^. The sequencing work was entrusted to Wuhan Frasergen Information Co., Ltd. (Wuhan, China).

### Genome annotation

RepeatMasker (v4.1.0, www.repeatmasker.org) and RepeatModeler (v2.0.1, https://github.com/Dfam-consortium/RepeatModeler) were used to annotate the repetitive sequences in the genome with default parameters. Infernal^[40]^ (v1.1.4) was used to identify noncoding RNAs (ncRNAS) by comparison with the RFAM database (v14.1). tRNAscan^[40]^ -SE (v2.0.7) was used to identify tRNA. RNAmmer v1.1.2^[41]^ was used to search for rRNAs. Funannotate (v1.7.2) was used for protein-coding gene prediction. EVidenceModeler (EVM, v1.1.1) was used to integrate the gene sets and to generate a relatively complete accurate Qu-1 gene structure annotation file.

### Genome visualization

CIRCOS (v0.69-9)^[42]^ was used to visualize various functional elements in the genome. Blastn (v2.10.1+) was used to self-align the Qu-1 genome nucleic acid sequence, integrate the adjacent alignment results, screen the collinearity blocks with a continuous length greater than 10 kb, and display them in the innermost circle of the genome circle map. A 10-kb window was used to calculate the GC content distribution of the Qu-1 genome. The further outward circle shows the noncoding region genes, the outer circle of noncoding genes displays the distribution of repetitive sequences, the outer circle displays the distribution of coding genes, and the outer circle represents the 19 bars of Qu-1 chromosome information.

### Identification of the SNV sites on the Qu-1 genome

To prevent interference of repetitive sequences and accurately identify the heterozygous sites on the Qu-1 genome, Bowtie2 (v2.4.5) was used with stringent parameters (--score-min L,0,-0.1 --end-to-end --no-unal --no-mixed --no-discordant -L 20 -i S,1,0.5 -N 0 --mp 6,2) to align NGS reads to the hardmasked reference genome, followed by the GATK (v4.3.0) pipeline for the SNV calling. The sites with sequencing depth less than 5 or more than 300 and heterozygous reads ratio of less than 20% or more than 80% were also filtered out to obtain high-quality SNV sites. We further manually inspected these SNV sites and selected 21 sites for PCR amplification and Sanger sequencing verification (primers are provided in Supplementary Table S1).

### Protoplast isolation and transfection of Qu-1

Protoplast isolation of the Qu-1 was performed by enzymatic hydrolysis methods as described previously^[17]^. The quantity and quality of protoplasts obtained were detected by using a blood cell counting plate. The pUC19-*35S*_*pro*_-*sGFP* vector encoding green fluorescent protein (sGFP) was used for transient transformation^[43]^, and the transformation sufficiency was estimated using Zeiss LSM700 laser-scanning microscopy. In addition, the pUC19-*35S*_*pro*_-*sGFP* vector was used for transient transformation, and GFP-positive cells were quantified by flow cytometry^[44]^. Protoplast transformation was performed by PEG-mediated transformation methods as previously described^[17]^. Bimolecular fluorescence complementation (BiFC) was performed with Qu-1 protoplasts to verify the interaction between PtrGCN5-1 and PtrAREB1-2, which are proteins related to drought inducibility and expressed abundantly in *P.* *trichocarpa* xylem tissue and are known to interact with each other^[45]^. Subcellular localization analysis (endoplasmic reticulum (ER), Golgi apparatus, mitochondria, peroxisomes, plasma membrane, plastids, tonoplast) was performed with the same system.

### *Agrobacterium* *tumefaciens*-mediated stable transformation of Qu-1

*Agrobacterium* *tumefaciens*-mediated transformation of the Qu-1 suspension cell line was performed as previously described in the research on tobacco (*Nicotiana* *tabacum*) BY-2 cells^[46]^. First, a single knockout mutant of the *PsnPDS* gene was created using the CRISPR/Cas9 system^[47]^. The single guide RNA (sgRNA) was designed based on the Qu-1 genome. The sgRNA sequence was synthesized and ligated into the pEgP237-2A-GFP vector. Then the *Agrobacterium*-*mediated* transformation method was used to stably transform Qu-1. The Qu-1 suspension cultured for 6 d was washed with 0.01 M PBS buffer (pH 7.2−7.4) 2−3 times. Then the Qu-1 suspension cells were transferred to the transformation solution containing *Agrobacterium* GV3101 strain (*Agrobacterium* GV3101 bacterial solution (OD_600_ = 0.2) + 0.75 mM acetosyringone (AS) + 0.0002 mg/L thidiazuron (TDZ), and then placed in a constant temperature shaker at 26 °C and 110 rpm for 12 h. The co-cultured Qu-1 cell suspension was centrifuged at low speed, the supernatant was removed, the cells were washed with sterile water, and the excess liquid and bacteria were removed by qualitative filter paper. Finally, the cells were resuspended with appropriate sterile water, and the cell suspension was evenly spread on the solid screening medium (MS + 1.25 mg/L 2,4-D + 0.2 mg/L KT + 50 g/L sucrose + 10 mg/L kanamycin + 100 mg/L timentin) with a sterile suction tube. After 25−30 d of dark culture, new cell clusters will appear. To detect the editing of the mutant, primers located on both sides of the sgRNA target sequence were designed for PCR amplification, and the PCR products were ligated into the pEASY-T1 vector (TIANGEN, Beijing, China). At least 40 colonies were selected for sequencing and the sequencing results were counted. The primers of *PsnPDS* gene are listed in Supplementary Table S1.

Using this method, the stable genetic transformation of over-expression and inhibition table of Qu-1 was carried out. The Qu-1 cell line was stably transformed by *Agrobacterium*-*mediated* transformation. The pROK2 vector carrying *PsnCYCD1;1* gene and the pRNAi vector carrying *PsnC3H3* were used as transformation plasmids. The DNA and transcription levels of the resistant cell clusters were detected, and the expression levels of other lignin monomer synthases in the transgenic plants (*RNAi*-*PsnC3H3*-*L15*) were detected by RT-qPCR. The primers used in the detection are shown in Supplementary Table S1.

### EMS mutagenesis Qu-1 and mutant site detection

Appropriate amounts of Qu-1 callus was placed in a mutagenic solution containing 0.05% EMS, and cultured at 26 °C, 110 rpm for 5 h. After that, the cell suspension was transferred to a 50 mL centrifuge tube, and Qu-1 cells were collected by centrifugation at 3,000 g for 4 min. The cells were resuspended with 40 mL ddH_2_O, and the supernatant was discarded by centrifugation at 3,000 g for 4 min. Finally, 2 mL ddH_2_O was added to the cells. The cells were transferred to MS medium (MS + 1 mg/L 6-BA + 0.2 mg/L NAA + 0.05 mg/L TDZ + 25 g/L sucrose + 3 g/L gelrite) using a dropper. After 30 d, the induced callus produced adventitious buds. Genomic de novo sequencing of mutagenic adventitious buds was carried out, and important mutation sites were determined by whole genome sequencing of EMS-induced buds. We selected 20 mutation sites for PCR amplification and Sanger sequencing verification (primers are provided in Supplementary Table S1). At the same time, six mutant genes were screened according to the mutation frequency, and the transcription level was detected. Reverse transcription of 1 μg of total RNA using a PrimeScript RT Kit with gDNA Eraser (Takara, RR047A). According to the manufacturer's instructions, TB Green *Premix* *Ex* *Taq* II (Takara, RR820L) was used for RT-qPCR detection and analysis on the Applied Biosystems7500 RT-qPCR system. The primers and gene information are listed in Supplementary Table S1.

## Results

### Characterization of the doubled haploid cell line Qu-1 from *P.* *simonii* × *P.* *nigra*

**Figure 1:**
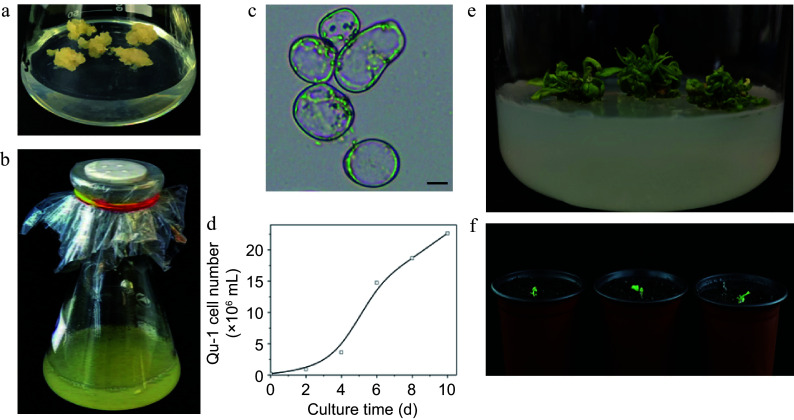
Growth characteristics of the doubled haploid cell line Qu-1 derived from poplar. (a), (b) Growth status of Qu-1 under solid culture and suspension culture conditions. (c) Microscopic observation of Qu-1 cell morphology. Scale bar = 10 μm. (d) Statistics of the number of cells per unit volume of Qu-1 under suspension culture conditions. (e) Clustered adventitious shoots obtained by inducing Qu-1. (f) Whole plants of Qu-1 obtained by induction.

**Figure 2:**
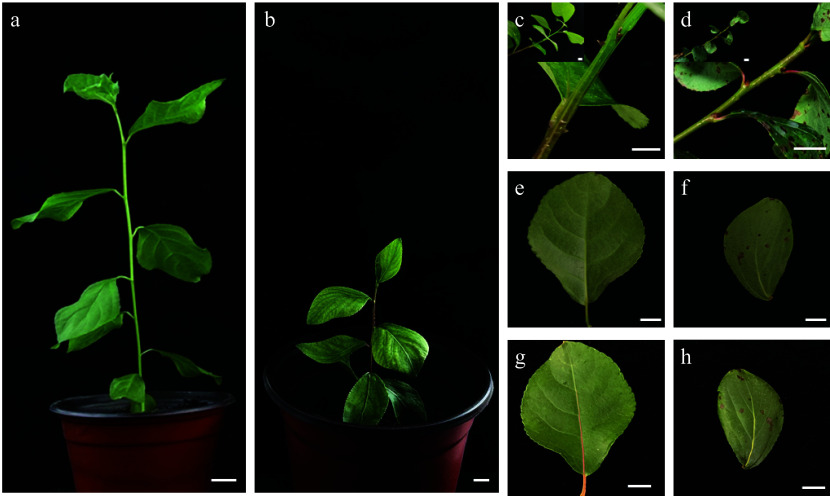
Analysis of phenotypic differences between Qu-1 and donor tree (DT) plants. (a) One-month-old DT plants cultured under greenhouse conditions. (b) Two-month-old Qu-1 plants cultured under greenhouse conditions. Scale bar = 1 cm. (c), (d) Stem segments of DT and Qu-1 plants, respectively. Scale bar = 1 cm. (e), (g) The lower and upper surfaces of leaves of DT plants, respectively. Scale bar = 1 cm. (f), (h) The lower and upper surfaces of leaves of Qu-1 plants, respectively. Scale bar = 1 cm.

We obtained more than 300 haploid callus lines through *in vitro* anther culture of the hybrid poplar *P. simonii* × *P. nigra*^[48,49]^. Subsequent screening using liquid suspension culture led to the identification of a unique doubled haploid cell line, which was named Qu-1 (Fig. 1a, b). Microscopic examination revealed that Qu-1 cells exhibited an exceptionally high degree of cellular dispersion under suspension culture conditions, with cellular morphology predominantly observed as individual cells or small cell aggregates throughout the observation field (Fig. 1c). When fluorescein diacetate (FDA) was used as a dye for cell activity detection, Qu-1 cells exhibited almost 100% viability (Supplementary Fig. S1a). Moreover, Qu-1 cells showed remarkable growth potential. After one week of suspension culture, the cell count of Qu-1 increased 103 times (Fig. 1d). After 20 d of suspension culture, the density of Qu-1 cells reached 8.5 × 10^6^ cells/mL, with an OD_600_ value of 2.00, which was 6.7 times higher than the BY-2 cells under the same culture conditions (OD_600_ = 0.299) (Supplementary Fig. S1b). To date, Qu-1 has been cultured in our laboratory for over 6 years, and the above suspension characteristics remain unchanged. Notably, Qu-1 retains its totipotent and can be induced to regenerate into complete plants (Fig. 1e, f). Compared to the donor tree (DT), Qu-1 plants displayed significant phenotypic differences. For instance, the height of 2-month-old Qu-1 plants was about half of the 1-month-old DT plants (Fig. 2a, b). The observation of stem morphology showed that the stem of the DT had ridges, while the stem of the Qu-1 plant was smooth and without ridges (Fig. 2c, d). In addition, Qu-1 plants had curly leaf surfaces, short petiole, and long oval leaf shapes. While the DT plants had flat leaf surfaces, medium-long petiole, and rhombic oval leaf shapes (Fig. 2e−h). These findings indicated that the doubled haploid cell line Qu-1 from poplar exhibited excellent suspension characteristics and could regenerate into complete plants, which provide new materials for molecular biology and genetics research.

### Ploidy analysis and generation of a high-quality genome assembly for Qu-1

**Figure 3:**
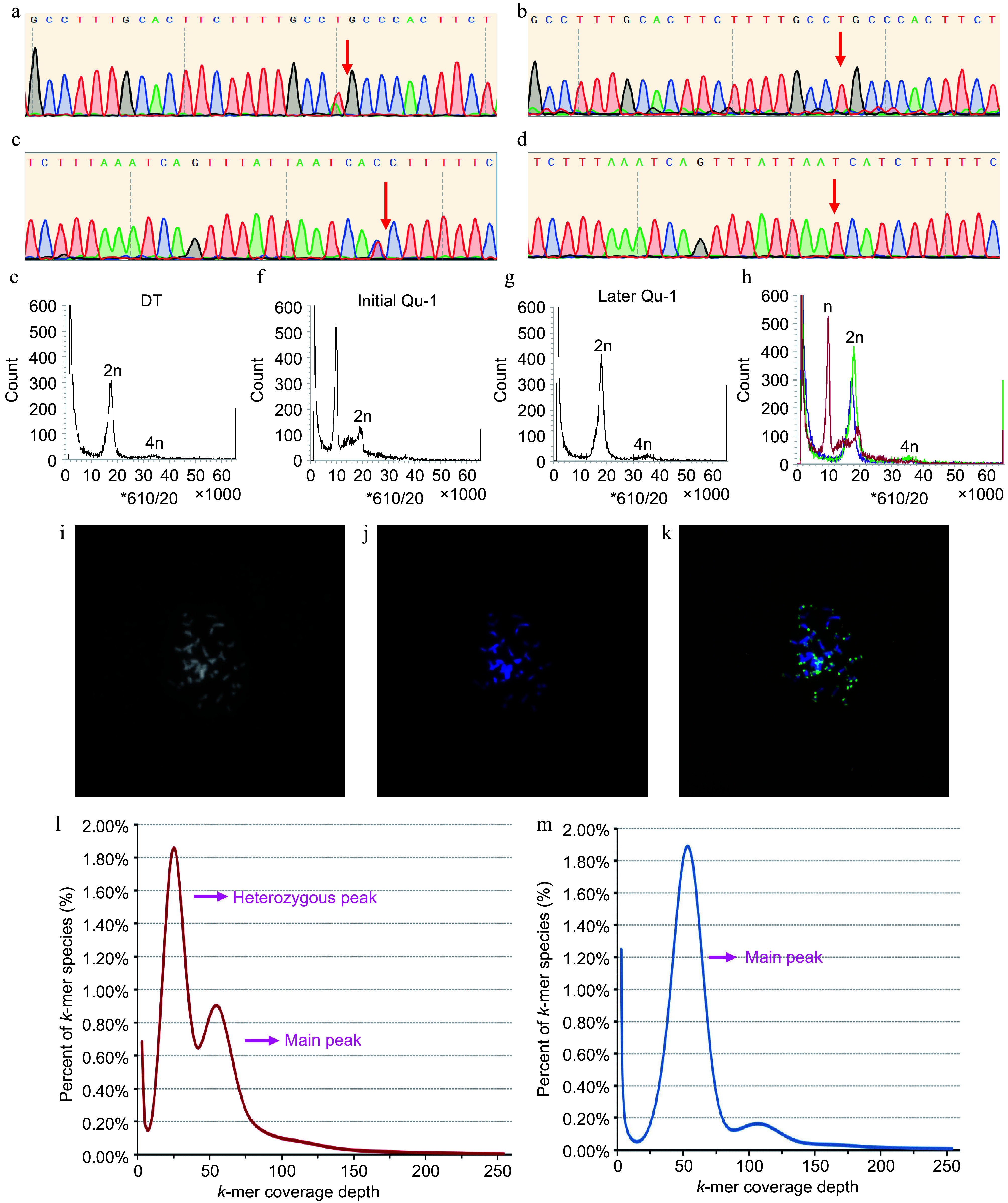
Homozygosity and ploidy analysis of Qu-1. (a)−(d) Homozygosity of Qu-1 and DT was detected by SNP-PCR method. (a), (c) Sequencing peaks of PCR products of DT with different specific SNP sites based on Sanger sequencing. (b), (d) Sequencing peaks of PCR products of Qu-1 with different specific SNP sites based on Sanger sequencing. The red arrows represent the SNP sites. (e) Ploidy analysis of diploid DT. (f), (g) Ploidy analysis of Qu-1 at early (i.e., just obtained), and late (i.e., current state) stages. (h) Merge of (e), (f), and (g). Red: represents the ploidy of Qu-1 at the early (i.e., just obtained) stage; green: represents the ploidy of Qu-1 at the late (i.e., current state) stage; blue: represents the ploidy DT. (i)−(k) Detection of chromosome number and structure of Qu-1 by the FISH method. 2n = 38. (i) Chromosome status of Qu-1 under bright field. (j) Fluorescence detection of chromosome status of Qu-1 after DAPI staining. (k) Detection of telomere distribution in Qu-1 chromosome using a telomere-specific probe. Blue represents DAPI-stained chromosomes, and green signals indicate telomere localization. (l) *k*-mer analysis of DT. (m) *k*-mer analysis of Qu-1.

We employed an SNP-PCR method, which combines specific SNP sites and PCR technology, to assess the homozygosity of Qu-1 and DT. The PCR products were subsequently subjected to Sanger sequencing. The results showed that DT displayed a double peak at the specific SNP site, indicating it is heterozygous, whereas Qu-1 displayed a single peak at this site (Fig. 3a−d). During the cultivation of Qu-1, to observe whether the ploidy of Qu-1 had changed, we used flow cytometry (FCM) to detect the DNA content of Qu-1. Interestingly, the DNA content of Qu-1 was half of DT at the early stage (immediately after obtaining), but after one year of culture, the DNA content of Qu-1 was the same with DT. The FCM results revealed a substantial change in the DNA content of Qu-1 during the culture process, transitioning from the initial haploid level to the doubled haploid level (Fig. 3e−h). Subsequently, we used fluorescence *in situ* hybridization (FISH) to further verify the chromosome number and structure of Qu-1. The FISH results revealed that Qu-1 had 38 chromosomes (2n = 38), indicating that Qu-1 experienced the doubling of the whole set of chromosomes during the culture process (Fig. 3i−k). This result was consistent with the results of FCM, which confirmed the ploidy change of Qu-1 cell line during culture. Furthermore, a *k*-mer analysis was performed on Qu-1 based on second-generation sequencing to estimate its genome size and genomic heterozygosity. For Qu-1, the *k*-mer distribution showed a clear main peak, suggesting that the genome had a homozygous source (Fig. 3m), while the *k*-mer distribution of DT showed a typical bimodal pattern consistent with the heterozygous genome of diploid plants (Fig. 3l), indicating that there was a high heterozygosity in the genome. Based on the total number of *k*-mer and the depth of the main peak, we estimated the genome size of Qu-1 to be about 403.7 Mb.

**Figure 4:**
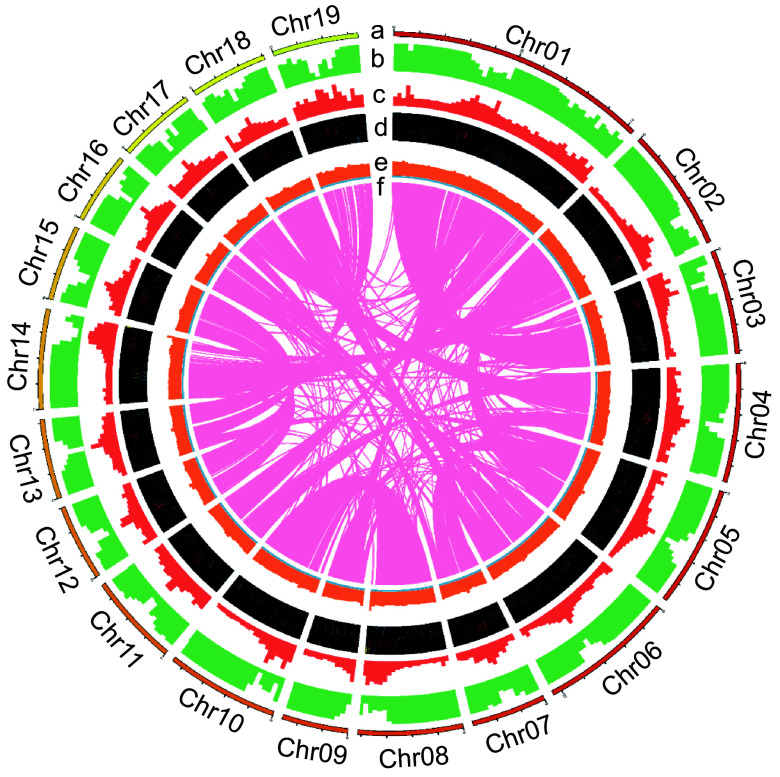
The chromosome distribution and annotated functional elements of Qu-1 are shown by the circle diagram. (a) Distribution of the 19 chromosomes of Qu-1. (b) Localization of coding genes on each chromosome. (c) Gene duplication patterns. (d) Distribution of rRNA, snRNA, tRNA, and miRNA genes. (e) GC content. (f) Homologous regions longer than 10 kb on the chromosomes.

To facilitate the application of Qu-1 in molecular biology, a chromosome-level genome was generated using PacBio long reads. The obtained assembly contained 327 contigs. The total length and N50 length of the assembly were 397.9 and 6.7 Mb, respectively (Supplementary Table S2). A total of 126 contigs were anchored to 19 pseudochromosomes using Hi-C reads, representing 97.3% of the total length (Supplementary Fig. S2, Supplementary Table S3). BUSCO assessment using the embryophyta_odb10 database indicated that 1,570 (97.3%) BUSCOs were fully covered, including 841 single-copy (52.1%) and 729 duplicated (45.2%), with 13 fragmented (0.8%) and 31 missing (1.9%). Genome continuity assessment showed that the adjusted LAI score of the genome was 16.3, within the range of 'reference' quality (10 ≤ LAI < 20). All these results indicated that the Qu-1 genome had high levels of accuracy, continuity, and completeness. Further annotation revealed that 49.3% of the Qu-1 genome was repetitive. A total of 37,331 protein-coding genes, 1,589 tRNA genes, 703 rRNA genes, 1,189 snRNA genes, and 747 miRNA genes were also identified in the genome. The distribution of chromosomes in the Qu-1 genome and the functional elements of the corresponding annotations are shown by the circle diagram (Fig. 4). To minimize false positives, we increased the stringency of our heterozygous single nucleotide variant (SNV) calling algorithm. In total, only 135 heterozygous SNV sites were successfully designed, 73 of which were located on chromosomes. However after manual examination, the majority of these sites are still located in highly-similar duplicated sequences. Of the 21 sites selected for PCR amplification and Sanger sequencing, none of them were proved positive (Supplementary Fig. S3). On the one hand, this proved that the Qu-1 had a very high homozygosity, and on the other hand, it also suggested that we should pay special attention to false positives when analyzing mutation sites in the future. In summary, we obtained high-quality genome sequence for Qu-1 at the chromosome level, which formed the foundation for subsequent studies.

### Establishment of genetic transformation systems for Qu-1

**Figure 5:**
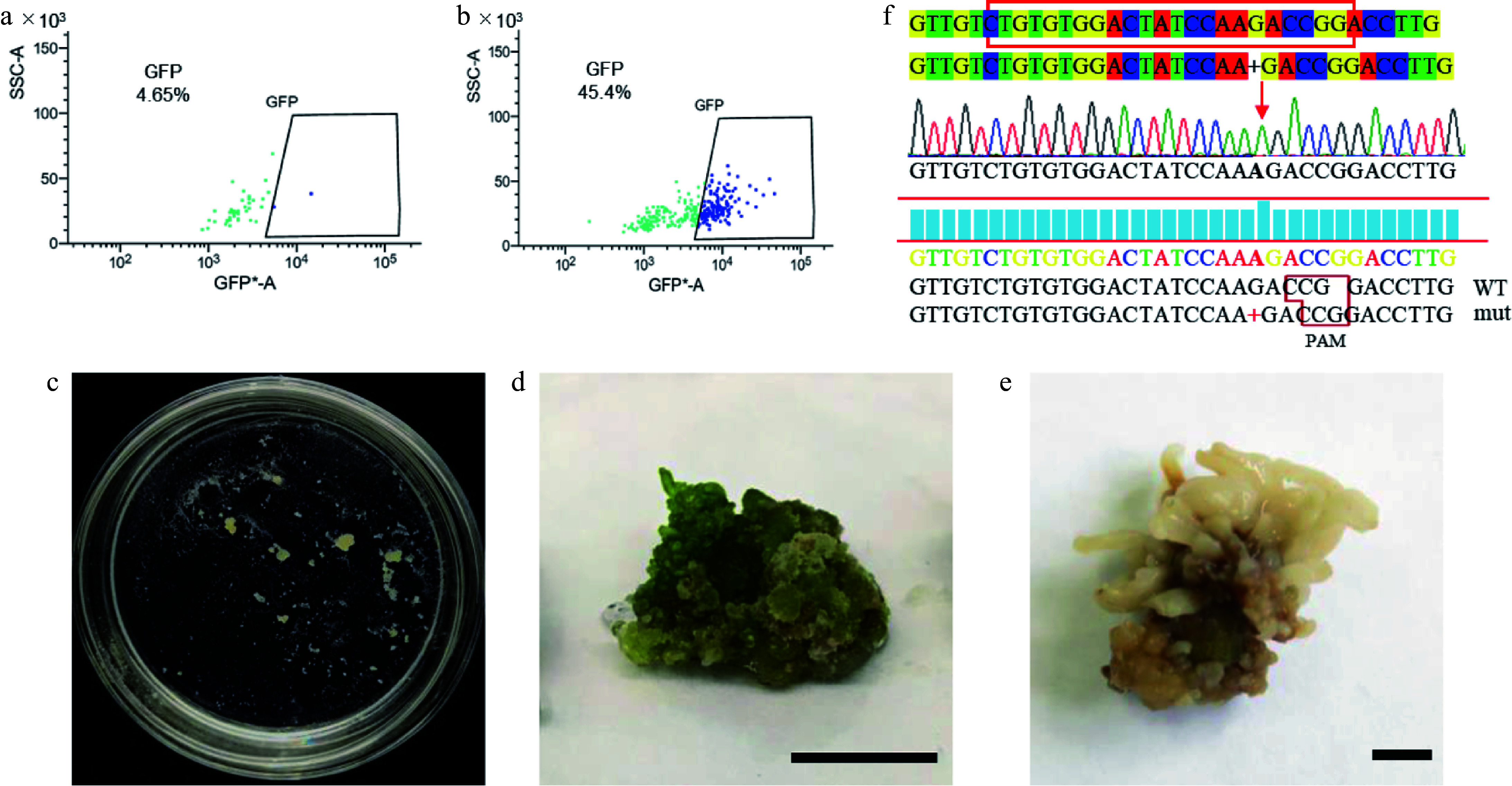
Transient transformation and stable genetic transformation of Qu-1 protoplasts. (a), (b) Analysis of Qu-1 protoplast transformation efficiency by flow cytometry. (a) Qu-1 protoplasts were used as a control. (b) PEG-mediated transient transformation of Qu-1 protoplasts with pUC19-*35S*_*pro*_-*sGFP* plasmid was analyzed by flow cytometry. (c) The acquisition of stable genetic transformation resistant callus by genome editing. (d), (e) Wild-type and *psnpds* mutants under light culture. Scale bar = 1 cm. (f) Sanger sequencing analysis of *psnpds* mutants.

Genetic transformation serves as a core method for analyzing gene function in molecular biology. Accordingly, we established a PEG-mediated protoplast transient transformation system for Qu-1. After the isolation of Qu-1 protoplasts, we introduced the pUC19-*35S*_*pro*_-*sGFP* vector plasmid into them using PEG-mediated method and then analyzed by flow cytometry and laser confocal detection. The results revealed that the transformation efficiency of Qu-1 protoplasts reached 40% (Fig. 5a, b; Supplementary Fig. S4a). We then performed BiFC to verify the interaction between PtrAREB1-2 and PtrGCN5-1 using the established transient transformation system^[45]^ and obtained a consistent result with the previous study (Supplementary Fig. S4b). Moreover, we utilized this system to achieve cellular localizations of organelles markers including endoplasmic reticulum, mitochondria, and Golgi apparatus (Supplementary Fig. S5). These results indicated that we successfully established a PEG-mediated Qu-1 protoplast genetic transformation system, making it a system suitable for gene expression analysis and subcellular localization analysis.

We then established an *Agrobacterium*-mediated stable genetic transformation system for Qu-1. We carefully screened the key parameters such as the growth status, bacterial concentration, infection time, and co-culture time of Qu-1. The results showed that a large number of transgenic experimental materials could be quickly obtained by mixing Qu-1 cultured for 10 d with low concentration of engineering bacteria and culturing for 12 h. Based on this optimization system, we further carried out a variety of genetic transformation experiments such as overexpression, RNA interference inhibition expression, and gene editing. Firstly, we used *Agrobacterium* *tumefaciens* containing pEgP237-PsnPDS-2A-GFP (GV3101) to transform Qu-1. Through resistance screening, resistant calluses were successfully obtained (Fig. 5c). The results of phenotypic observation showed that these resistant calli showed albino phenotype under light culture compared with the wild type (Fig. 5d, e). DNA molecular detection and Sanger sequencing analysis further confirmed the successful acquisition of the mutant (Fig. 5f). In addition, we also carried out stable genetic transformation experiments of overexpression and RNA interference inhibition. The *Agrobacterium* (GV3101) containing pROK2-*PsnCYCD1;1* and pRNAi-*PsnC3H3* was used for genetic transformation of Qu-1, and molecular detection was performed at the DNA and RNA levels. The results showed that the transgenic lines with overexpression and inhibition of expression were successfully obtained (Supplementary Figs S6a−c & S7a−d). Because the *PsnC3H3* gene is one of the key enzymes in lignin monomer synthesis, we analyzed the expression levels of other lignin monomer synthases in RNAi-suppressed transgenic lines (*RNAi*-*PsnC3H3*-*L15*) by RT-qPCR. The results showed that compared with the wild type, the expression of lignin monomer synthase gene *PsnCCR2*, *PsnCAD1*, *PsnCSE1*, *PsnCSE2*, *PsnCCoAMT1* and *PsnCCoAMT2* in the transgenic lines was inhibited, while the expression of *PsnPAL5* and *PsnHCT1* remained basically unchanged, and the expression of other lignin monomer synthase genes was significantly increased (Supplementary Fig. S7e). In summary, Qu-1 has established an efficient protoplast transient transformation system and a stable genetic transformation system including genome editing, which lays a solid foundation for becoming a powerful tool for molecular biology research.

### The high-quality genome of Qu-1 facilitates the identification of mutation sites in Qu-1 induced by EMS

**Figure 6:**
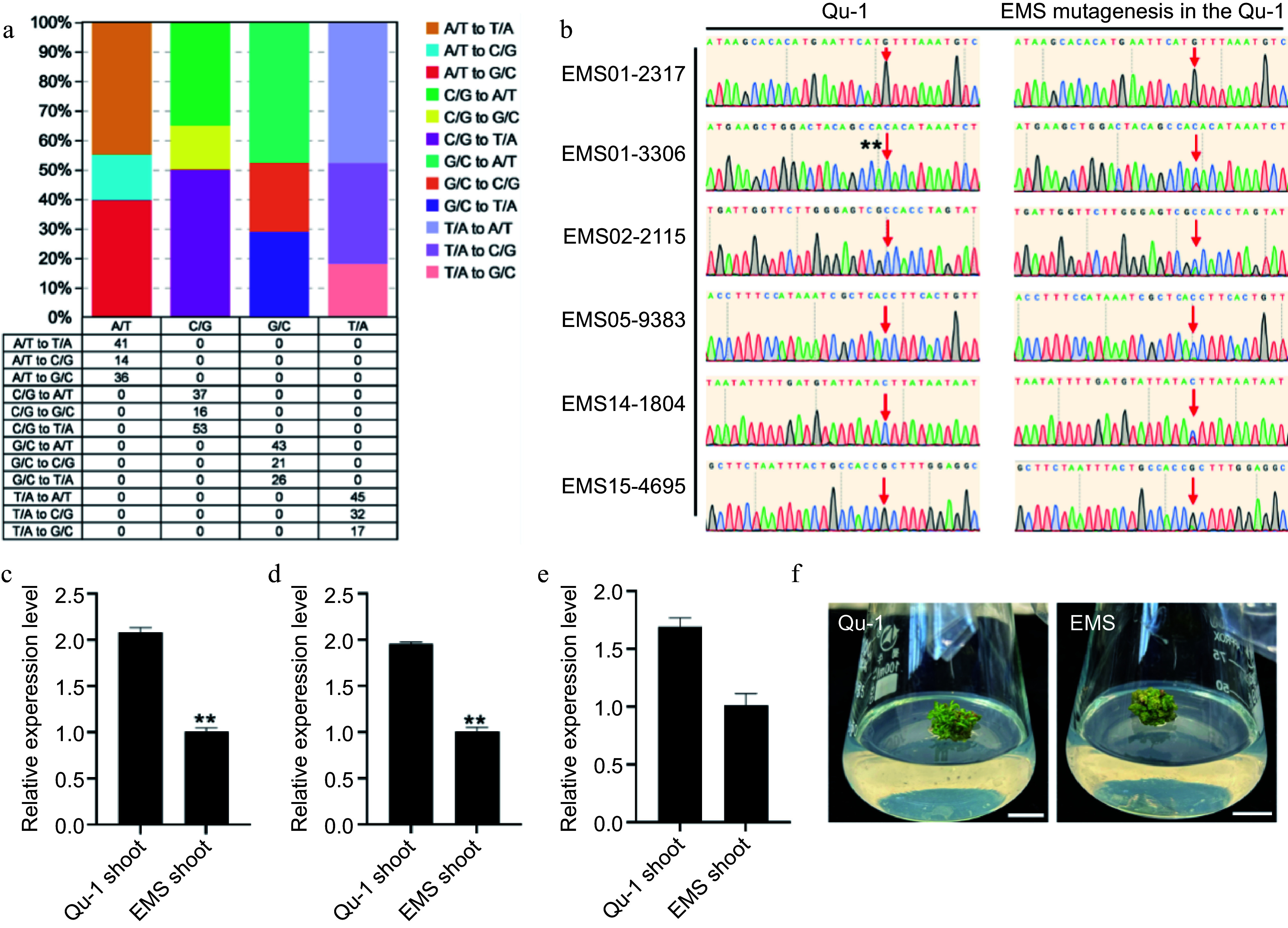
Detection of SNV sites and phenotypic observation of EMS-induced Qu-1 mutants. (a) The number and proportion of SNV sites induced in the EMS mutant genome. (b) Sanger sequencing detection of SNV sites EMS01-2317, EMS01-3306, EMS02-2115, EMS05-9383, EMS14-1804, and EMS15-4695 of EMS mutants. (c)−(e) Transcription abundance of mutant genes *QuPop00389*, *QuPop03514*, and *QuPop15257*. Error bars represent SE values of three biological replicates. Asterisks indicate significant differences between Qu-1 and EMS mutant plants by Student's t-test (**, *p* < 0.01). (f) Phenotypic observation of EMS mutants.

To verify the potential of Qu-1 as a genomic research tool, we performed EMS mutagenesis on Qu-1 callus and re-sequenced the mutagenic materials to identify the mutation sites. Our analysis revealed 200 predicted EMS-induced single nucleotide variation (SNV) sites, with an average of 0.25 SNV per million base pairs. Among these SNVs, C/G to T/A conversion was the most common, accounting for 50.0%, followed by A/T to T/A conversion (45.05%), C/G to A/T conversion (34.91%), and A/T to G/C conversion (39.56%). In addition, we also observed less A/T to C/G transversion (15.38%) and C/G to G/C transversion (15.09%). The ratio of conversion and transversion was 0.81 (Fig. 6a). Through PCR detection of 20 randomly selected SNV sites, we found that the sequencing results of PCR products of 16 sites were positive (Fig. 6b; Supplementary Fig. S8). Further mutation frequency annotation helped us to identify 21 mutant genes and quantitatively analyze the transcription levels of these genes. The results showed that the transcription levels of seven genes (*QuPop00389*, *QuPop03514*, *QuPop14948*, *QuPop18982*, *QuPop15257*, *QuPop26889,* and *QuPop29187*) in EMS mutants showed a downward trend (Fig. 6c−e; Supplementary Fig. S9). By observing the development status of EMS mutants, we found that the development rate of EMS mutants was significantly slower than that of wild-type (Fig 6f), which was similar to that of PPD5 (homologous to *QuPop15257*) mutants in *Arabidopsis* *thaliana*^[50]^. The above results indicate that Qu-1 is an effective genomic research tool that can generate heritable mutations through EMS mutagenesis, and is identified and verified by resequencing and molecular biology methods. In addition, these variations can affect the transcription level of genes and the development process of plants, further confirming the potential application value of Qu-1 in gene function research and genetic improvement.

## Discussion

The development of an efficient and high-quality suspension cell line is crucial for molecular biology research. In this study, we successfully established a doubled haploid cell line, Qu-1, from *P.* *simonii* × *P.* *nigra*, which exhibits superior characteristics for suspension culture and has the potential to regenerate into whole plants. The acquisition of this cell line, along with its high-quality genome sequence, marks a significant advancement in the field of molecular biology research on woody plants. Our findings reinforce the value of suspension cultures as a foundational tool for plant molecular and genetic studies.

The Qu-1 cell line demonstrated exceptional growth in suspension growth, with nearly 100% cell viability and a rapid cell proliferation. This robust performance is essential for various molecular biology applications, including gene expression analysis and functional genomics^[7,9]^. Moreover, the totipotency of Qu-1, as evidenced by its ability to regenerate into whole plants, is a valuable trait for studying plant development and genetics. Although the regenerated plants showed significant phenotypic variation compared to the donor tree. Such a variation is likely attributable to genetic changes during the haploid induction and cell culture. Similar phenotypic variations have been reported in other studies of haploid or doubled haploid plants^[48]^. The ability to regenerate plants from Qu-1 provides a means for genetic analysis and improvement of poplar.

The high heterozygosity in the genomes of many plant species, including poplar, has been a significant obstacle in molecular biology and breeding studies^[19]^. The high homozygosity of Qu-1, as indicated by the SNP-PCR method and *k*-mer analysis, is a significant advantage for genetic studies, as it reduces the complexity of genome analysis and facilitates the identification of causal genes for specific traits^[18,20]^. The high-quality chromosome-level genome assembly of Qu-1, achieved using PacBio long reads, resulted in a high continuity and completeness genome, which is essential for accurate gene annotation and functional studies. The low number of heterozygous SNV sites identified in Qu-1 further confirms its high homozygosity, making it an ideal model for genetic transformation and gene function analysis^[21,22]^. EMS is a chemical widely used to induce mutations in plants, which generates genetic resources for identifying and characterizing genes to understand the molecular basis of important agronomic traits. However, the random locations in trees induced by EMS hardly could be identified by genome sequencing due to their high heterozygosity. The ability of Qu-1 to generate heritable mutations through EMS mutagenesis and the subsequent identification of mutation sites by resequencing further demonstrate its potential as a genomic research tool. The observed mutation frequency and types are consistent with previous studies on EMS mutagenesis in plants. The identification of mutant genes and their associated phenotypic changes provide insights into the genetic basis of plant development and stress responses.

We also established efficient genetic transformation systems for Qu-1, including PEG-mediated protoplast transient transformation and Agrobacterium-mediated stable genetic transformation. These systems allow for rapid and precise gene manipulation, which is essential for functional genomics and molecular biology research^[1,19]^. The successful application of these transformation systems in Qu-1 highlights its potential as a powerful tool for studying gene function in woody plants.

## Conclusions

In this study, a doubled haploid cell line, Qu-1, derived from *Populus* *simonii* × *P.* *nigra*, was successfully established, which exhibits excellent suspension culture characteristics and totipotency. Using SNP-PCR and *k*-mer analyses, we verified that Qu-1 is highly homozygous, providing a valuable tool for woody plant research. With high-quality genomic sequences assembled using PacBio long reads and the development of both transient and stable transformation systems, Qu-1 is expected to facilitate genetic and molecular research, highlighting its utility in plant biology and biotechnology applications. Taken together, our results demonstrate that Qu-1 is a powerful tool for studying and advancing our understanding of woody plant biology and supporting applications in genetic improvement and biotechnology.

## SUPPLEMENTARY DATA

Supplementary data to this article can be found online.

## Data Availability

The whole genome sequence data and the annotation in this article can be found in the China National Center for Bioinformation database (www.cncb.ac.cn) (ID: PRJCA038715).
