# Supplementary material for: Qu-1: a transformation-and regeneration-amenable doubled haploid cell line with a reference genome sequence for genetic and functional studies in Populus
Source: For Res (Fayettev). 2025 Apr 29;5:e008. doi: 10.48130/forres-0025-0008 (PMC12141832; doi:10.48130/forres-0025-0008)
Supplement: Supplementary file 1 — Supplementary data to this article can be found online. [file forres-0025-0008-Supplementary.zip › 10.48130_forres-0025-0008-Suppl-FigureS1.pdf]

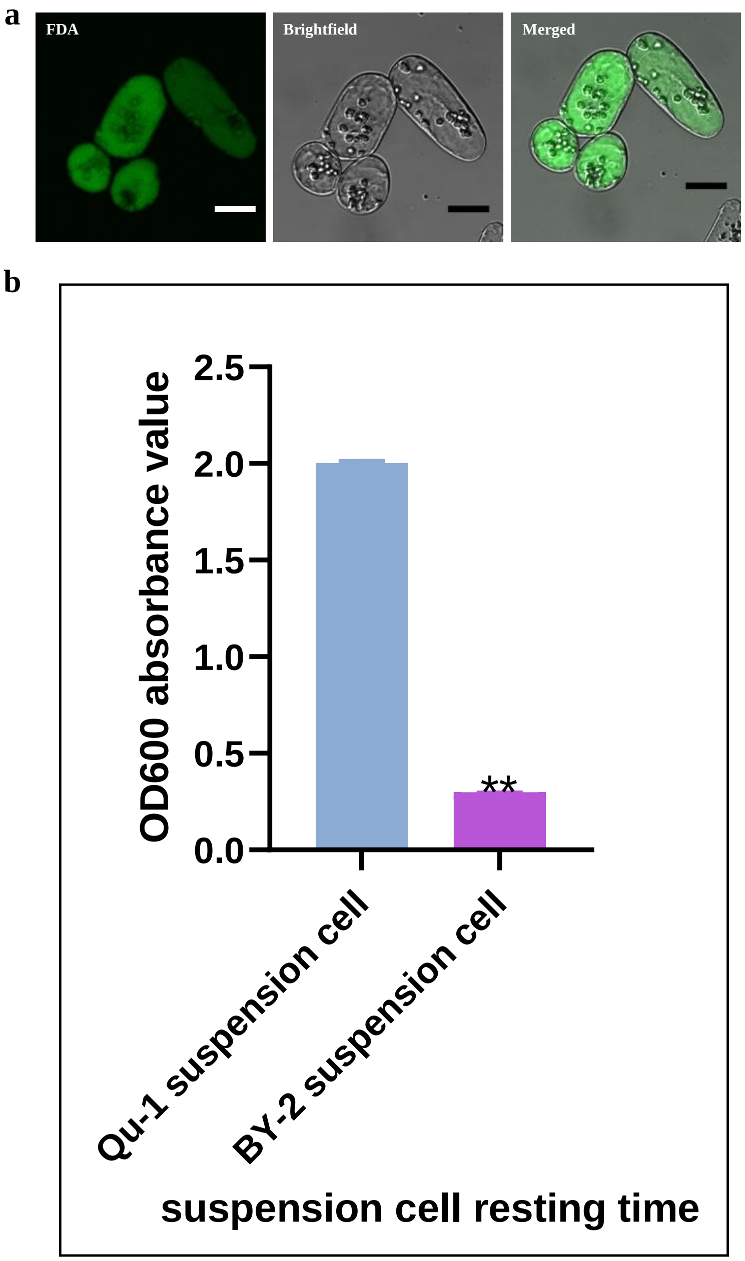

Supplementary Fig. S1

Characteristics of Qu-1 suspension cell line.(a). Qu-1 cells also exhibited almost 100% viability using Fluorescein diacetate (FDA) as a probe. Bar=20 μm ; (b). After 20 days cultured of Qu-1and BY-2 cells in medium reached an absorbance of OD600. Asterisks indicate **P<0.01 (Student's t-test).
