# Supplementary material for: Qu-1: a transformation-and regeneration-amenable doubled haploid cell line with a reference genome sequence for genetic and functional studies in Populus
Source: For Res (Fayettev). 2025 Apr 29;5:e008. doi: 10.48130/forres-0025-0008 (PMC12141832; doi:10.48130/forres-0025-0008)
Supplement: Supplementary file 1 — Supplementary data to this article can be found online. [file forres-0025-0008-Supplementary.zip › 10.48130_forres-0025-0008-Suppl-FigureS3.pdf]

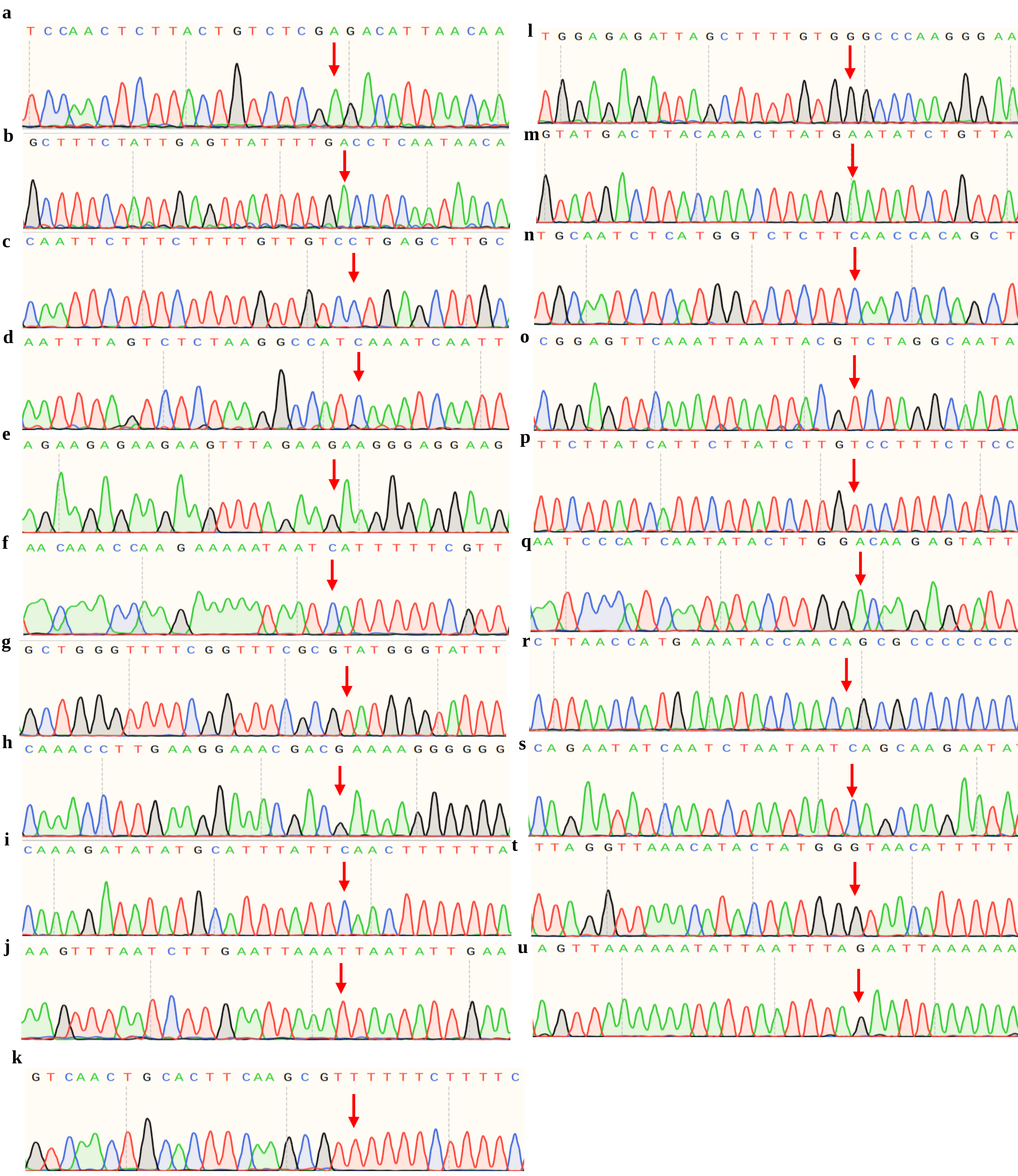

Supplementary Fig. S3

21 SNV sites for PCR amplification and Sanger sequencing of Qu-1. (a). SNV sites Chr02-11295484. (b). SNV sites Chr04-180016000. (c). SNV sites Chr05-12601504. (d). SNV sites Chr07-4292160. (e). SNV sites Chr08-9578323. (f). SNV sites Chr09-10703442. (g). SNV sites Chr11-1821092. (h). SNV sites Chr11-5224484. (i). SNV sites Chr11-5569835. (j). SNV sites Chr13-7593775. (k). SNV sites Chr14-9364405. (l). SNV sites Chr15-7563453. (m). SNV sites Chr01-15602766. (n). SNV sites Chr02-1623577. (o). SNV sites Chr03-991809. (p). SNV sites Chr05-18706325.(q). SNV sites Chr07-14456431.(r). SNV sites Chr08-5530890. (s). SNV sites Chr17-13384126. (t). SNV sites Chr18-13414161. (U). SNV sites Chr19-13199854.
