# Supplementary material for: Qu-1: a transformation-and regeneration-amenable doubled haploid cell line with a reference genome sequence for genetic and functional studies in Populus
Source: For Res (Fayettev). 2025 Apr 29;5:e008. doi: 10.48130/forres-0025-0008 (PMC12141832; doi:10.48130/forres-0025-0008)
Supplement: Supplementary file 1 — Supplementary data to this article can be found online. [file forres-0025-0008-Supplementary.zip › 10.48130_forres-0025-0008-Suppl-FigureS6.pdf]

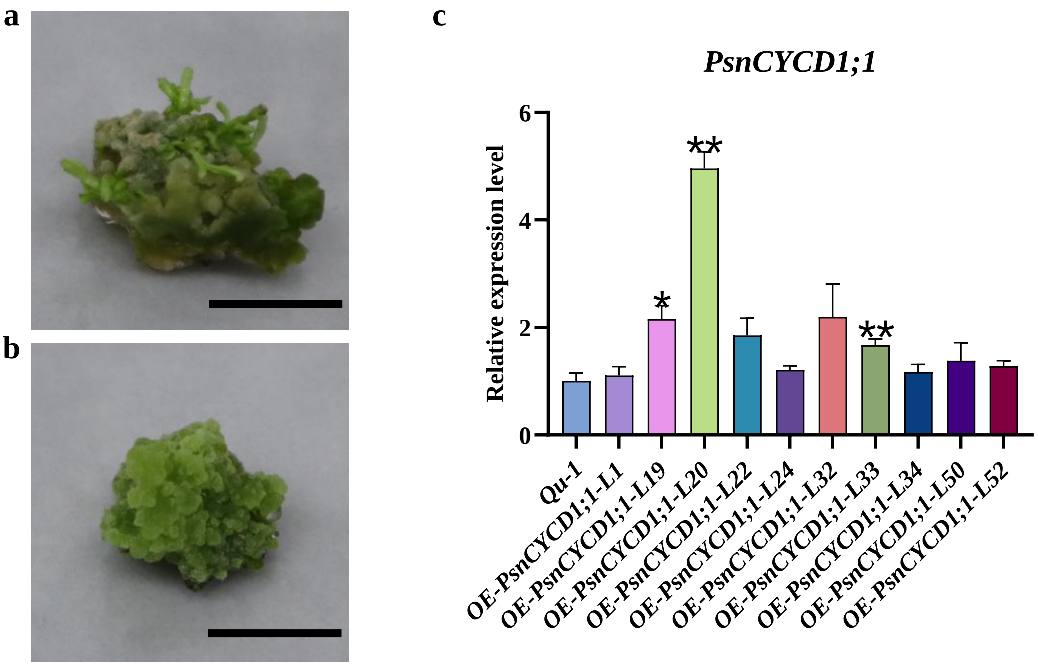

Supplementary Fig. S6

Molecular detection of pROKII-*PsnCYCD1:1* transgenic callus. (a-b). In vitro regeneration of Qu-1 plants expressing pROKII-*PsnCYCD1:1*, (a) Transgenic cell line , (b). Wild type. (c). RT-qPCR for RNA level detection of transgenic Qu-1(pROKII-*PsnCYCD1:1*). Asterisks indicate *P<0.05, **P<0.01 (Student’s t-test).
