# Supplementary material for: Qu-1: a transformation-and regeneration-amenable doubled haploid cell line with a reference genome sequence for genetic and functional studies in Populus
Source: For Res (Fayettev). 2025 Apr 29;5:e008. doi: 10.48130/forres-0025-0008 (PMC12141832; doi:10.48130/forres-0025-0008)
Supplement: Supplementary file 1 — Supplementary data to this article can be found online. [file forres-0025-0008-Supplementary.zip › 10.48130_forres-0025-0008-Suppl-FigureS7.pdf]

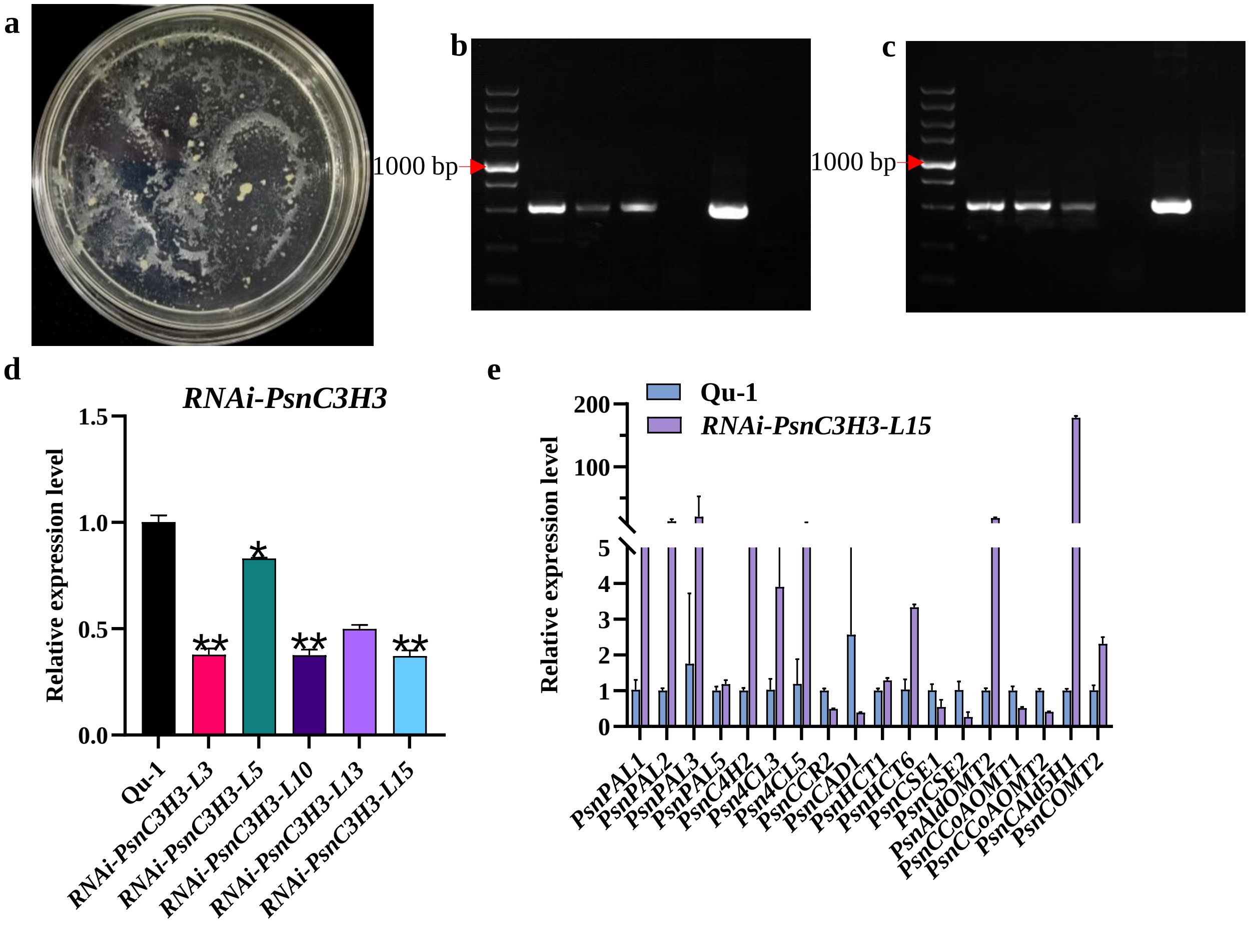

Supplementary Fig. S7

Molecular detection of pRNAi-PsnC3H3 transgenic callus. (a). Acquisition of kanamycin-resistant callus after culture for 4 weeks. (b-c). PCR for DNA level detection of transgenic Qu-1. (b). pRNAi-*PsnC3H3* transformed Qu-1DNA detection (35S-intro); (c). pRNAi-*PsnC3H3* conversion to Qu-1 DNA detection (35S-intro-Tnos). (d). qRT-PCR for RNA level detection of transgenic Qu-1(pRNAi-*PsnC3H3*).(e). Analysis of lignin monomer synthase gene expression levels in transgenic Qu-1 (pRNAi-*PsnC3H3*). Asterisks indicate *P<0.05, **P<0.01 (Student's t-test).
