# Supplementary figures and images for: Qu-1: a transformation-and regeneration-amenable doubled haploid cell line with a reference genome sequence for genetic and functional studies in Populus
Source: For Res (Fayettev). 2025 Apr 29;5:e008. doi: 10.48130/forres-0025-0008 (PMC12141832; doi:10.48130/forres-0025-0008)

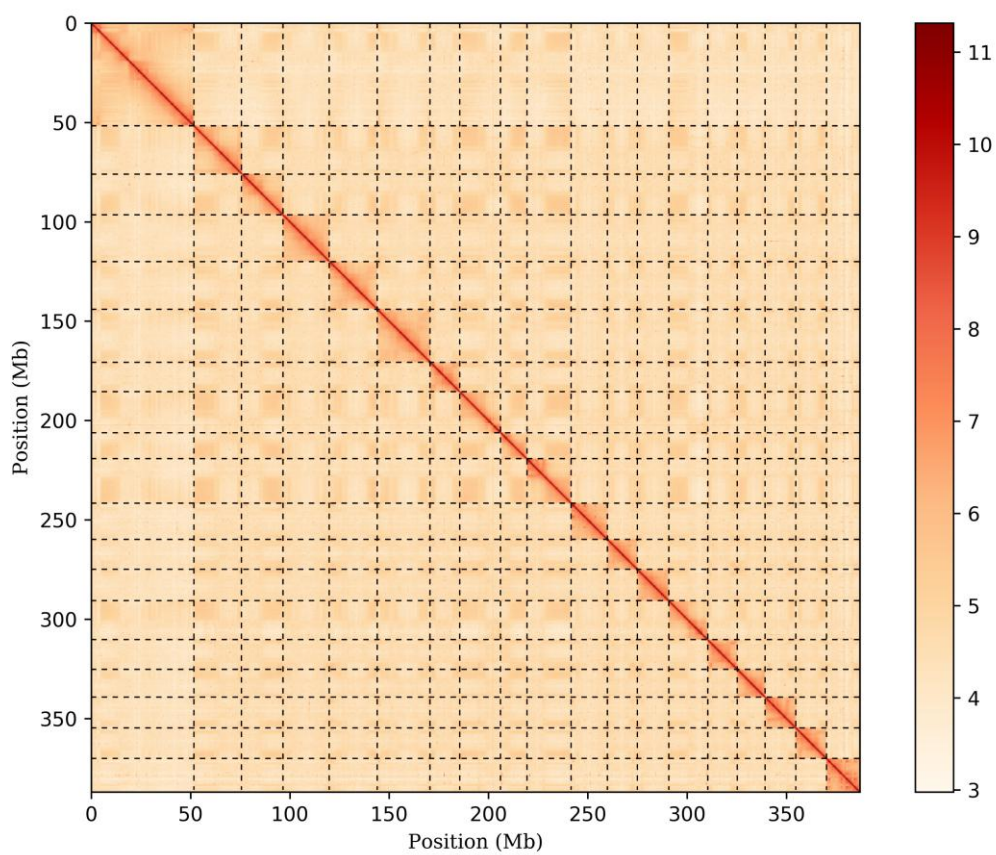

## Supplementary Fig. S2

Interaction frequency distributions of Hi-C linkage groups.

Supplement: Supplementary file 1 — Supplementary data to this article can be found online. [file forres-0025-0008-Supplementary.zip › 10.48130_forres-0025-0008-Suppl-FigureS2.pdf]
